# Improving data use in decision-making and utilization of maternal healthcare services through a data-informed platform for health approaches in districts of the Gedeo Zone, southern Ethiopia, 2023: a cluster-randomized control trial

**DOI:** 10.3389/frhs.2023.1125399

**Published:** 2023-08-21

**Authors:** Getachew Assefa Zenebe, Wagaye Alemu, Daniel Yehualashet, Mequanint Nakachew

**Affiliations:** ^1^ School of Public Health, College of Medicine and Health Sciences, Dilla University, Dilla, Ethiopia; ^2^ Department of Midwifery, College of Medicine and Health Sciences, Dilla University, Dilla, Ethiopia

**Keywords:** implementation research, data use, data-informed platform for health, maternal health service, Ethiopia

## Abstract

**Background:**

In low-resource countries such as Ethiopia, the utilization of local data for planning and decision-making health systems was frequently constrained. In addition, despite several government initiatives, maternal health services were not completely utilized. On the other hand, efforts to effectively utilize the local data available to improve the utilization level of maternal healthcare services were insufficient, necessitating the need for a different approach.

**Objective:**

This implementation study aims to test and validate the effectiveness of a data-informed platform for health (DIPH) strategies on data use for decision-making and utilization of maternal health services in districts of the Gedeo Zone, southern Ethiopia.

**Methods:**

A two-arm parallel group, type II hybrid, cluster-randomized control trial design has been implemented to conduct the study between 1 September 2022 and 29 February 2024. Six woredas/districts have been assigned to the intervention arm and the other six to the control arm. Baseline and end-line data have been collected from 120 eligible health management staff (from both intervention arm and control arm). In the intervention arm, district health management staff have been given specialized training and continuous technical assistance as a package called the DIPH strategy by embedding it with the district's current decision-making platform such as Performance Review Team meetings. The DIPH strategy has mainly focused on five-step approaches such as situational assessment, stakeholder engagement, defining/setting priorities, planning, and follow-up. Health management staff in the control arm have performed their regular daily activities. The *χ*^2^ and *t*-tests have been used to check the effect of the intervention. In addition, difference-in-differences estimates have been calculated because the change may inherently occur over time. A *P*-value of <0.05 and a 95% confidence interval have been used to declare the significance of the intervention.

**Discussion:**

The findings of this study were supposed to give insights into implementation strategies that improve data use in decision-making and utilization of maternal healthcare services at the woreda level and uncover contextual factors that boost the response of these strategies.

## 1. Introduction

Management and problem-solving capacity at all levels are essential for establishing effective and efficient health systems and expanding access to basic healthcare services in resource-limited settings. Effective decision-making is particularly vital in low-income settings, where scarce resources must be carefully stewarded and efficiently deployed to meet the substantial health needs of the population (1, 2). Effectively and efficiently managing the six components of the health management information system (HMIS), namely, indicators, data sources, data administration, information products, distribution, and usage, is possible (3).

At present, maternal and child health (MCH) care services [antenatal care (ANC) services, delivery, postnatal care (PNC) services, and so forth] are the most prioritized service areas that need critical decisions globally and require special attention to reduce maternal mortality and morbidity to achieve sustainable development goals (SDGs) (4). Therefore, a standardized and widely employed approach or strategy that increases the use of locally available data is needed to improve the performance of those services. As a result, the data-informed platform for health (DIPH) is one of the approaches aimed at improving data use and mother and child health (5).

The DIPH is a straightforward procedure for district-level decision-making based on health data and would aid in addressing local community needs better (5). DIPH is a structured decision-support approach aimed at encouraging the use of locally available data in health decision-making at the district level. With the systematic use of data by district administrative and program leaders for health program prioritization and planning, progress monitoring, and follow-up across various health domains (such as maternal, newborn, and child health), the adoption of the five-step DIPH approach—situational assessment, stakeholder engagement, defining/setting priorities, planning, and follow-up—has simplified the utilization of local-level program management and health service data. A method for including diverse stakeholders in structured health decision-making at the district level is included, which is already in place (5–7).

In low-resource countries such as Ethiopia, the utilization of local data for health system planning and decision-making is frequently constrained (7). The HMIS generates huge amounts of data regarding health service delivery and population health, enabling decentralized health systems to make data-driven decisions. Health facilities are trying to utilize local data available in health sectors (health post, health center, and hospital), such as daily, weekly, monthly, quarterly, and annually reported services and diseases, as well as data from other sectors such as nutrition, water, and sanitation, and other data directly related to the improvement of maternal health services (4). However, the data are rarely used locally (5). In practice, HMIS data are underutilized at the community and district levels because there is no defined process for their utilization, or they may be unavailable, incomplete, or of poor quality (8, 9).

Improving health information systems will initially result in improved outputs of higher quality and timely data. Then, data must be utilized to improve the functioning of the five other building blocks of the health system, namely, health services, the workforce, medical supplies, financing, and leadership to finally affect service delivery and health outcomes. However, data are frequently insufficiently utilized in program creation and improvement, policy formulation, strategic planning, or advocacy and instead sit on reports, shelves, or databases (8).

According to the literature, the overall level of routine data or health information used for decision-making among health workers working in health institutions is insufficient and varies by country and region of the world. In some African countries other than Ethiopia, it runs from 38% in Côte d'Ivoire to 69.6% in Kenya (10–14). The result of a systematic review in Ethiopia revealed that the overall utilization of data among care providers was 57.42% (15), which ranges from 45.8% in the East Gojjam Zone to 78.5% in North Gondar (16–20).

Evidence suggests that if locally available data are not well utilized, decisions to improve the performance of major health services may be distorted, contributing significantly to their low achievement (7, 17, 21). According to global SDGs (4) and the strategic plan of the Ethiopian health sector (22), maternal health services are identified as a prioritized area. Information revolution is among the five transformation agendas, and the utilization level for those services was low. For example, in eastern African countries, the utilization rate of at least four or more antenatal healthcare services stood at 52.4%, of which 31.82% is from Ethiopia (23). The Ethiopia Mini-Demographic and Health Survey–2019 (EMDHS-2019) shows 74% and 43% of mothers used first and fourth ANC services, respectively. Regarding delivery, 50% were attended by a qualified practitioner, and 48% occurred at a healthcare facility. Moreover, 34% of women received a PNC check-up within the first 2 days after birth, and 41% used modern methods of family planning (FP) (24).

Many factors contributed to the low level of data utilization and performance of maternal healthcare services, including organizational and external factors and a lack of a well-established approach. Evidence is provided for a limited number of procedures, including using locally available data for district-level decision-making. Much has been published about the collection of healthcare data and measures to improve data quality, but how the data are used is less well documented (5, 25).

Standardization and pre-testing strategies in various scenarios would increase the potential of being widely employed. DIPH, which organizes woreda-level decision-making using health data, would help meet the needs of the local community, requiring standardization in our context (5).

Although our country has implemented several plans to enhance health management information systems and maternal and child health services, they have not been achieved as expected (22). Because of this, a structured and well-developed approach, which has the potential to improve the HMIS and MCH services, is very important.

To the knowledge of the researchers, no implementation study in Ethiopia assesses the effects of the DIPH strategy on data use in decision-making and utilization of maternal and child health care services at the woreda level. Therefore, interventional research with a comparison group is necessary to ascertain if the reported benefits are attributable to the evaluation awareness of the personnel of the facility, other secular trends, or impact of the legitimate intervention.

Using the data gathered from this study, managers can establish suitable strategies to address challenges linked to data use and maternal healthcare at the woreda level. Stakeholders such as regional, zonal, and woreda health management staff, program focal persons from each health facility, and other health workers can employ this strategy to improve the utilization of data and maternal health services and scale up to a broader context.

As a result, the current implementation research intends to evaluate the effects of the DIPH strategy on data use in decision-making and utilization of maternal health services in districts in the Gedeo Zone of southern Ethiopia in 2022.

### 1.1. Outcome

• Improved data use in decision-making among health management staff in districts through the DIPH strategy.
• Improved utilization level of maternal health services (antenatal care, delivery, postnatal care, and FP services) in districts through the DIPH strategy.

### 1.2. Research questions

• What will be the effect of the DIPH approach on data use in decision-making?
• What will be the effect of the DIPH approach on the utilization of maternal health services (ANC, delivery, PNC, and FP)?

## 2. Methods and materials

### 2.1. Study setting

This study has been conducted in Gedeo, one of the administrative zones in the Southern Nations, Nationalities, and Peoples’ Region (SNNPR), Ethiopia. According to the 2007 Ethiopian Population and Housing Census, the total population of the Gedeo Zone was estimated to be 130,433, with an area of 1,210.89 km^2^ (26). Currently, the zone has a total population of 1,086,769 [532,515 (49%) male and 554,224 (51%) female] (27).

The Gedeo Zone has 12 woredas, including Dilla Town, Kochore, Chelelektu Town, Gedeb Town, Chorso, Yirga Chefe Town, Yirga Chefe woreda, Dilla Zuria, Gedeb woreda, Wonago, Bule, and Rappe. It has one referral hospital, three primary hospitals, 38 health centers, 146 health posts, and nine non-governmental organization (NGO) clinics, and there are private health facilities, including low-level clinics, medium clinics, drug stores, and pharmacies that provide health services for the community. Data from the Gedeo Zone health department have estimated the health service coverage to be 92% (28). This implementation study has been conducted in all woredas of the Gedeo Zone by allocating six of them to the intervention arm and six to the non-intervention arm.

### 2.2. Study design and the implementation period

This implementation research has been conducted using a two-arm parallel group, type II hybrid, cluster-randomized control trial (C-RCT) design in the Gedeo Zone woredas. The study has taken 18 months (four cycles with 3–4 months each) including two pre- and post-data collection months (between 1 September 2022 and 29 February 2024). The intervention (DIPH approach) has been provided for district-level management staff as a package, which includes DIPH training, DIPH job aid, and implementation support.

### 2.3. Study participants

The study participants have been the head of the health department, all program officers, and data managers at the woreda level. All eligible woreda staff have been interviewed. We have anticipated that 10 eligible staff members will be available in each woreda, and we have intended to include all of them in the study.

### 2.4. Eligibility

#### 2.4.1. Inclusion criteria

All woredas of the Gedeo Zone and all managerial and administrative staff working in those facilities between 1 and 30 September 2021, pre-intervention, and 1–29 February 2024, post-intervention, have been included in the study. The interventions have been provided from 1 October 2022 to 30 January 2024.

#### 2.4.2. Exclusion criteria

Study participants who have not given consent and those who have worked in their respective woreda for less than 6 months have been excluded.

### 2.5. Determination of sample size

The sample size estimation for the required number of woredas per study arm is based on the statistical formula recommended by Hayes and Bennett (29), assuming 80% power and at least 25 percentage point difference in the performance of data use and maternal healthcare services.

$$c=1+(z_{\alpha /2}+z_{\beta }{)}^{2}\frac{\pi_{0}(1-\pi_{0})/m+\pi_{1}(1-\pi_{1})/m+(k_{0}^{2}\pi_{0}^{2}+k_{1}^{2}\pi_{1}^{2})}{{\left(\pi_{0}-\pi_{1}\right)}^{2}}$$

where *C* is the number of clusters required (woredas), *Z_α_*_/2_ is the confidence level (95%), Z*_β_* is required power (80%), π_0_ is the true proportion in the absence of intervention (50%), π_1_ is the true proportion in the presence of intervention (75%), *m* implies the individuals sampled in each cluster/woreda (10), *k*_0_ is the coefficient–variation of true proportion in the absence of intervention (0.096942), and *k*_1_ is the coefficient–variation of true proportion in the presence of intervention (0.096942).

**Table:** 

Indicator	Expected level at baseline	Expected minimum percentage increase	Health management staff assessed/woreda	Number of woredas in study arm
Level of data use in decision-making at the woreda level	50%	25%	10	6
Maternal health service utilization	50%	25%	10	6

Based on the calculations above, a minimum sample size of 120 health management staff (60 per study arm) has been required to conduct the study.

### 2.6. Selection of the population and allocation of the intervention

**Figure 1:**
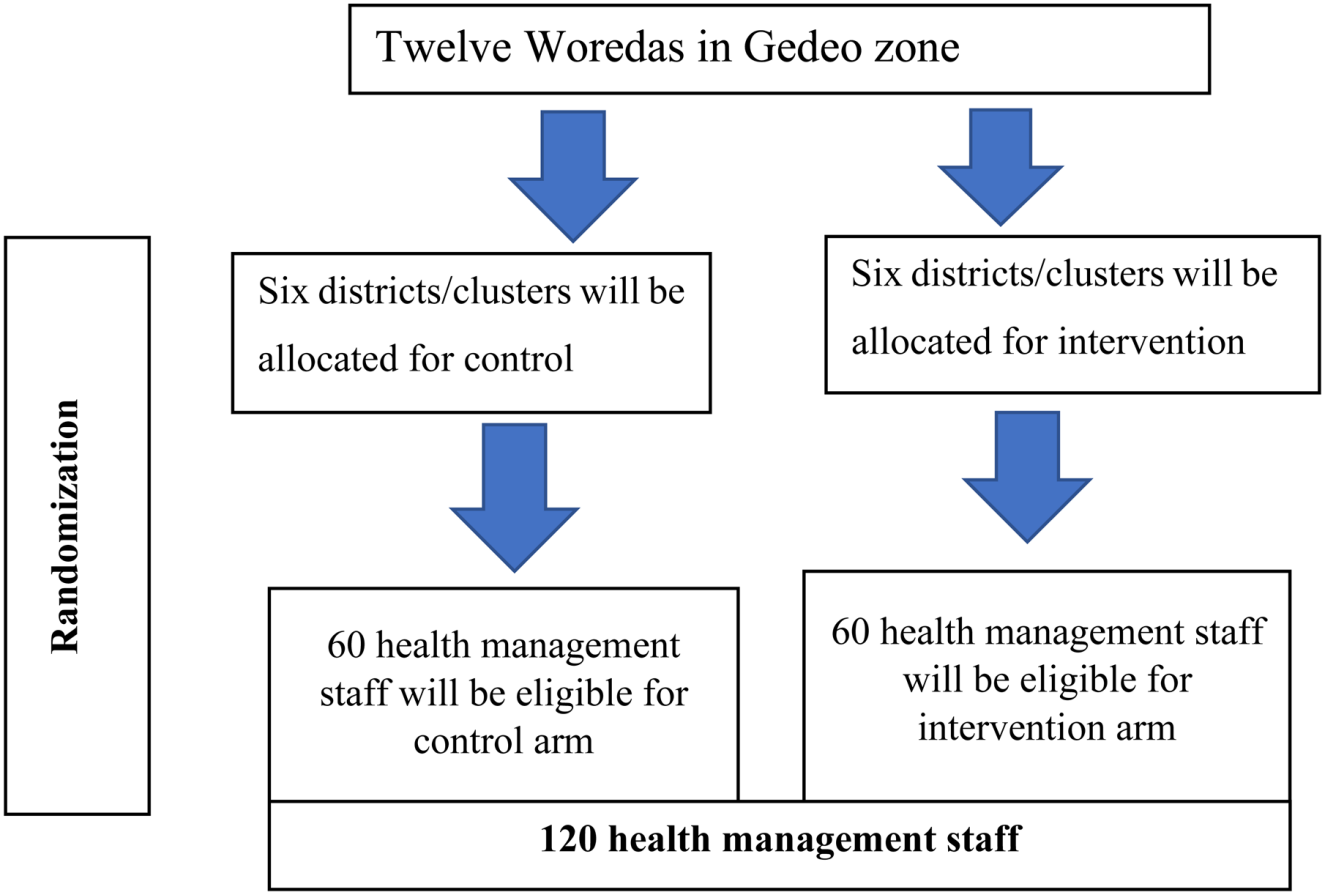
Sampling procedure on improving data use in decision-making and maternal healthcare service utilization through DIPH approach in districts, Gedeo Zone, southern Ethiopia, 2022.

The implementation research has been conducted in all woredas of the Gedeo Zone. Study woredas have been treated as clusters, with an equal number allocated to both intervention and non-intervention arms. Matched peer random allocation has been employed. To reduce subjective allocation, matching has been performed based on the performance level of the woredas (based on key indicators) and distance. The woredas have also been classified as urban and rural areas, and six intervention and six comparison arm woredas have been randomly selected in collaboration with the zonal health department. To avoid information contamination between the intervention and control arms, a buffer zone has been created based on the distance within the districts. Finally, all health management staff have been included in the selected woredas (Figure 1).

#### 2.6.1. Embedding

The DIPH approach has been provided as a package and integrated into the current decision-making platform of the districts [such as Performance Review Team (PRT) meetings], adding a formal and structured coordination process between various departments and data sharing for evidence-based decision-making, planning, and resource allocation in accordance with local health priorities. The PRT, sometimes called the Performance Monitoring Team (PMT), which is a multidisciplinary team primarily responsible for improving data quality and using information regularly to evaluate progress and enhance healthcare delivery, is the main platform for routine data use in the health sector. In Ethiopia, the PRT or PMT is led by the head of the facility. Department leaders and/or case team leaders serve as members of the HMIS office, which acts as a secretary. The PRT meetings of the woreda health management have been used as a platform to introduce the DIPH packages.

Activities have been primarily focused on

• encouraging the regular analysis and use of available data to comprehend woreda-level health system improvement.
• strengthening the PRT forum to engage all government and non-governmental stakeholders in identifying challenges and solutions, as well as assessing resource allocation and responsibilities. These meetings have been convened with the aim of consensus building and collective decision-making to improve health systems and services in their areas.

### 2.7. Blinding

• The nature of the interventions has been designed for implementation by management health workers. As a result, it cannot mask program implementers and health workers. However, during implementation, baseline, and endpoint data collection, all study participants and data collectors would be kept in the blind about the research question and hypothesis that the team came up with.

### 2.8. Phases of the implementation

The study has four core phases, such as the orientation phase, preparatory phase, intervention phase, and monitoring and evaluation phase.

#### 2.8.1. Orientation/discussion phase

*Policy dialog*. Before commencing implementation, including its training phase, a detailed discussion has been conducted with responsible bodies on the potential implementation of the strategies in the zone. Stakeholders include the zonal health department, program managers, technical experts, and non-governmental organizations; finally, a consensus has been achieved among them.

#### 2.8.2. Preparatory phase

*Prepare materials***.** Logistics, including job aids such as guidelines, has been prepared before the implementation.

*Organizing the DIPH field team*. The researcher team, also referred to as the implementation support team, has consisted of six members, including one health professional each from health service management, health informatics, reproductive health, epidemiology, and biostatistics and one health management staff from the zonal health department as a coordinator, supervisor, and data manager. To facilitate the overall activities, the principal investigator of the research is responsible. The field team has the following roles: prepare and develop data collection tools, provide training to health professionals, and conduct monthly supportive supervision using a performance checklist in line with the health facility PRT. The teams will acquire and examine data from six district health offices, which are DIPH-implementing districts, and six control districts that will not be participating in DIPH. According to the priority areas determined by the district health offices, the PRT evaluates monthly progress toward a set of indicators.

The assistance provided by the DIPH team, which consists of action plan preparation, follow-up, and supportive supervision, benefits the team leaders of the district health office. As the purpose of the DIPH is to strengthen decision-making, unit leaders of district health offices are working with the DIPH field team to gain a clearer understanding of their data and make better use of it.

#### 2.8.3. Implementation/intervention phase

*Training*. Before initiating the first DIPH cycle, the district health management staff of DIPH woredas has been provided 5 days of intensive training based on the designed curriculum in their zonal city. On average, 10 health administrative staff per woreda have received training from the research team. The training also involves experts from the zonal health department, in addition to the head of the health department of woredas, program officers, and an HMIS focal person. The DIPH research investigator team has prepared the 5-day residential training. DIPH-related issues, including the strategy concept, training guidelines, and other evidence, have been clearly discussed with each stakeholder during the workshop. Then, readjustment has been performed in accordance with the discussion and recommendations raised by the workshop participants, and the study areas/districts have been selected together with those invited participants.

*Technical support*. Those woreda health managers will benefit from the organized implementation support team throughout the intervention. The intervention will focus on five basic steps of DIPH (assess, engage, define, plan, and follow-up). This strategy enabled the health management staff to assess the situations based on the available data, engage stakeholders, prioritize the identified problems, and prepare a clear monthly action plan and follow-up.

#### 2.8.4. Monitoring and evaluation phase

1. To ensure the quality of the intervention provided and data collected, the research team and other stakeholders have had frequent meetings to share progress, best practices, challenges, and possible solutions to overcome barriers to the implementation.
2. Monitoring and evaluation of the intervention activity have been assessed from the beginning to the end of the intervention. Both qualitative and quantitative methods have been used in the evaluation. Depending on the findings of each monitoring and evaluation, necessary modifications and adjustments have been made to the intervention progress. We have used formative and summative evaluation during and following the initiation of the intervention.
3. The performance of the DIPH strategy has been reviewed and evaluated during monthly supervision and quarterly meetings, at which point necessary feedback has been given. The parameters for this assessment will be the number of Performance Monitoring Team meetings conducted, problems identified and prioritized, action plans prepared, and monitoring and evaluations conducted. In addition, potential obstacles/barriers have been identified and discussed during the meeting. Woredas with better performance have been appreciated and recognized, and their experience has been shared with other woredas for better implementation results.
4. Finally, the intervention effect on data use in decision-making and utilization of maternal healthcare services has been assessed at the end.

### 2.9. Operational definition

**The DIPH approach** is a structured decision-support approach in promoting the use of locally available data for health decision-making at the woreda level (7).

**Data use in decision-making** is a process through which district health managers, decision-makers, and stakeholders explicitly consider the information in gap identification, prioritization, root cause analysis, action plan development, and follow-up. The five components of the outcome variable were identifying indicators in the department, calculating targets vs. achievements, providing feedback to health workers at the lower levels, calculating program coverage, and evidence showing the use of data to notify decisions. It has been measured by computing 20 yes/no questions and said to be good if all the responses are yes unless poor (30, 31).

**Maternal health service utilization** is the overall utilization of common maternal healthcare services such as antenatal care, delivery, postnatal care, and family planning from a provider standpoint (report) in percent. It has been measured using 10 core indicators, each at 100% (21, 32).

### 2.10. Data collection tools and procedures

A pre-tested interviewer-administered structured questionnaire, based on the Performance of Routine Information System Management tools (version 3) (30) and adapted to the local context, has been used to improve data use in decision-making and utilization of maternal healthcare services through the DIPH approach in districts of the Gedeo Zone, southern Ethiopia, 2022. The questionnaire has two broad categories, namely, data use in decision-making and utilization of maternal healthcare service. It will be organized in an English version and then translated into local languages (Gedeuffa and Amharic) and finally retranslated into an English version to ensure consistency. The collection of baseline data has been carried out by the organized field teams. For end-line survey data collection, an independent team of four data collectors and one supervisor has been recruited for 1 month.

### 2.11. Data quality assurance

Before starting data collection, 5 days of training has been given to data collectors and the supervisor on the basic techniques of data collection and supervision. The supervisors have checked the accuracy, consistency, and completeness of the data daily. The questionnaire has been pre-tested by the same data collectors on 5% of the sample size (five health management staff) at one of the nearby district health offices with similar characteristics.

### 2.12. Data management

The data already collected have been routinely checked for completeness and consistency. After appropriate cleaning and coding, the data have been entered into EpiData version 4.6 and exported to SPSS version 23 software for analysis. Descriptive statistics have been generated and presented in narrative, figurative, and tabular forms. The *χ*^2^ and *t*-tests have been used in checking the statistical significance of the intervention and the difference in data use in decision-making and utilization of maternal healthcare services within and between groups. We have employed difference-in-differences estimates because the change may inherently happen over time. Therefore, a control group has captured this intrinsic temporal change without DIPH intervention and subtracted it from the change brought by the DIPH intervention to assess the net effect on key study outcomes. We have calculated the absolute differences in percentages from the baseline and the end line, then calculated the net effect by subtracting the absolute DIPH intervention difference from the control. A *P*-value of <0.05 and 95% confidence interval (CI) have been used to declare the significance of the intervention.

### 2.13. Ethical consideration

An ethical approval letter was obtained from the Institutional Review Board of Dilla University (Ref. No.: duchm/irb/026/2023), and an official letter of cooperation request (Ref. No.: 1000/206/15) was submitted to the respected health facilities. After discussing and explaining the purpose and whole course of the study, written informed consent has been obtained from each health administrative staff member just before the actual data collection. Confidentiality has been assured at all stages of the study by making the questionnaire anonymous. Participant involvement has been voluntary, and those who have been unwilling and needed to quit their participation in the interim have been informed of their right to do so.

### 2.14. Dissemination plan of the result

The findings of the study have been presented to Dilla University and national and international research conferences. The result of the research has been disseminated to the zonal health office and respective health facilities. It has been prepared to be published in a reputable and peer-reviewed journal. This study has been scaled-up if the effectiveness of the intervention is assured.

## 3. Discussion

In low-resource countries such as Ethiopia, the utilization of local data for planning and decision-making health systems is frequently constrained. In addition, despite several government initiatives, maternal health services were not completely utilized. Therefore, this implementation research has been the key priority area in the healthcare system, and it has attempted to standardize possible policy strategies which emanate from multi-level contextual factors. This implementation research has attempted to develop novel insights into how the local government program implements using the established approach. The findings of this study are supposed to give insights into implementation strategies for policymakers and stakeholders to improve data use in decision-making and utilization of maternal healthcare services at the woreda level and to uncover contextual factors that boost the response of these strategies.
