# Supplementary material for: Improving data use in decision-making and utilization of maternal healthcare services through a data-informed platform for health approaches in districts of the Gedeo Zone, southern Ethiopia, 2023: a cluster-randomized control trial
Source: Front Health Serv. 2023 Aug 21;3:1125399. doi: 10.3389/frhs.2023.1125399 (PMC10475933; doi:10.3389/frhs.2023.1125399)
Supplement: Supplementary file 1 [file Table1.docx]

**Annex: Log Frame Matrix**

**Table 1**: Log Frame Matrix improve data use in decision-making and maternal health care service utilization through DIPH approach in districts, Gedeo Zone, Southern Ethiopia., 2022.

| Description of the Interventions | OVI (Objectively verifiable indicators) | Data Sources | Assumptions |
| --- | --- | --- | --- |
| **Goal:**  Improving maternal health care service utilization | **Outcome** -Percentage of Maternal healthcare services utilized (ANC, Delivery, PNC, FP) | -DHIS_2_ reports  -KPI reports  -Registrations of  maternal healthcare services | -If the trained professional stay at the district and work as to the standard  -If all the required equipment, essential drugs, and supplies are sustained |
| **Objective:** Improving data use in decision making | **Outcome**-The proportion of health management staff who utilize data for decision-making properly | -Data use survey checklist |  |
| **Strategies:**  DIPH package: DIPH job aid, Training and Implementation support by focusing on:   - Assessing the situation - Engaging relevant stakeholders, - Defining priorities, - Developing action plans - Following-up of action plans. | **Outputs**  -Number of health management staff who received DIPH training  -Number of supportive supervision and mentorship on DIPH conducted  -Number of written feedbacks provided after the mentorship  -Number of employees selected as best employee of the month/rewarded. | -HRIS staff training profile  -Observation and  inventory of tangible resources  -IFRR (Intra facility report and  request) form  -Supportive supervision and mentoring checklists |  |
| Activities   - Communicating and conduct policy dialog with ZHD and Dilla University research team - Preparing training materials and other necessary materials - Providing the training on Data informed platforms for health approach/strategy packages - Develop TOR on regular supportive supervision providing through implementation support team - Perform regular and intensive supportive supervision and mentorship/technical assistance - Conduct a discussion with staff and provide written feedback after a mentorship - Motivate high-performer employees and health facilities on regular bases | | | |
